# Orchids reduce attachment of herbivorous snails with leaf trichomes

**DOI:** 10.1371/journal.pone.0285731

**Published:** 2023-08-18

**Authors:** Richa Kusuma Wati, Barbara Gravendeel, Rob Langelaan, Bertie Joan van Heuven, Jean Claessens, Jacques Kleynen, Erik F. Smets, Anton J. de Winter, Arie van der Meijden

**Affiliations:** 1 Naturalis Biodiversity Center, Leiden, The Netherlands; 2 Research Center for Biosystematics and Evolution, National Research and Innovation Agency (BRIN), Cibinong, Bogor, Indonesia; 3 Institute of Biology Leiden, Leiden University, Leiden, The Netherlands; 4 Radboud University, Radboud Institute for Biological and Environmental Sciences, Nijmegen, The Netherlands; 5 Evolution and Biodiversity Conservation, KU Leuven, Ecology, Heverlee, Belgium; 6 BIOPOLIS Program in Genomics, Biodiversity and Land Planning, CIBIO, Campus de Vairão, Vairão, Portugal; William & Mary, UNITED STATES

## Abstract

Protective structures in the epidermis are essential for land plants to defend themselves against herbivores. In this study, we investigated the effect of different types of trichomes of three orchids, *Calanthe triplicata*, *Dendrochilum pallidiflavens* and *Trichotosia ferox*, on attachment of herbivorous land snails, using histochemistry and centrifuge experiments. Size, ornamentation and histochemistry of epicuticular trichomes on the orchid leaves were assessed with light microscopy, scanning electron microscopy and transmission electron microscopy. Total forces needed to detach two differently shaped snail species, *Subulina octona* and *Pleurodonte isabella*, were measured using a turntable equipped with a synchronized strobe. Snails were placed in two positions, either perpendicular or parallel to the main veins on the orchid leaves, both on the adaxial (= upper) or abaxial (= lower) side. The results obtained provided three new insights. First, a perpendicular or parallel position of the snails to the main veins did not significantly affect the attachment performance of either species tested. Secondly, snails detached significantly easier on leaf sides covered with a high density of lignin filled epicuticular trichomes. Thirdly, the removal of glandular trichomes did not affect the attachment forces; however, the absence of lignified trichomes increased the attachment of the snails. Our study highlights the importance of studying micro-ornamentation in combination with performance for obtaining a better understanding of the defense mechanisms employed by different species of orchids to deter herbivorous snails.

## Introduction

The surface of sessile organisms like plants plays a crucial role in environmental interactions [1]. Understanding the ecological and evolutionary interactions of plants and herbivores has been a subject of interest for many decades. Plants evolved different strategies to avoid consumption by herbivores. First of all, they have a physical barrier, either through development of structures such as waxy cuticle, trichomes, spines and setae or producing raphides in their tissue [2–7]. Secondly, they can defend themselves chemically by producing secondary metabolites [8–10]. Thirdly, they often evolved a symbiosis with natural enemies of herbivores such as ants [11–13].

To feed on a plant, small herbivores need to attach themselves to a plant’s surface [14]. Plant physical defensive structures are often embedded in the epidermal cells [1]. Examples of such protective structures are trichomes. Trichomes are hair-like appendages extending from the epidermis that can be straight, spiral, hooked, branched or unbranched, and glandular or non-glandular [15]. Trichomes can be a deterrent due to their mechanical and/or toxic effects [3]. Trichomes in high densities interfere with the movements of small herbivores on a plant, thus reducing access to the leaf surface [2]. Secondary metabolites secreted by glandular trichomes can be poisonous, repellent, or even trap insects, thus forming a combination of structural and chemical defense [3, 6].

Orchids are among the largest families of flowering plants. Although mostly known for their spectacular floral diversity, orchids show substantial anatomical diversity of the leaves as well [16, 17]. Despite the large variation in epi- and subcuticular protective structures, orchids suffer from herbivore damage, both in the wild as well as in cultivation, which may be particularly detrimental to many endangered species in nature [18] and causes huge annual capital losses to orchid nurseries worldwide [19]. Common invertebrate orchid pests are slugs and snails [20].

Compared to the many publications on orchid-insect herbivore biology and ecology [21–24], little has been published on understanding orchid-snail herbivory. Terrestrial snails and slugs adhere to and traverse many types of surfaces by using a thin layer (~10–20μm) of mucus secreted by the sole surface [25–27]. To propel themselves, they create a series of pulses by muscles in the foot that interact with the substrate through mucus secreted by the animal [28, 29]. Shirtcliffe et al. [30] hypothesized that the amphiphilic (possessing both hydrophilic (water-loving) and lipophilic (fat-loving) properties) nature of the mucus plays an important role in adhesion of snails to many different types of surfaces. These authors based their hypothesis on the fact that they were able to reduce snail adhesion using a weak surfactant (a compound that lowers the interfacial tension between a liquid and a solid) that changed the wetting response of the surface of plant pots to the sole surface. The mucus layer helps the snail to create a stable attachment to any substrate at various inclinations [29]. Adhesive locomotion on a smooth surface is less costly in terms of mucus production as compared to a rough surface [31]. A recent study of Krings et al. [32] showed that the radula is also involved in increasing mechanical interlocking with a substrate while feeding on a rough or irregular surface.

During fieldwork in tropical and temperate regions over the past twenty years, we observed that some orchid species are much more affected by herbivory than others (Gravendeel pers. obs.). We hypothesize that this phenomenon is caused by multiple factors, and that one of these factors involves leaf ornamentation acting as a deterrent to herbivores, possibly affecting detachment forces in the field. To test our hypothesis that orchid leaf ornamentation is involved in anti-herbivore defense, we investigated (i) the leaf anatomy and histology of three orchid species with trichomes of different type, density and length with Scanning Electron Microscopy (SEM), Light Microscopy (LM), and Transmission Electron Microscopy (TEM), (ii) adhesion of high spired-Subulinidae and low-spired Pleurodontidae snails in relation to the presence of different epicuticular properties: non-glandular lignified trichomes and glandular trichomes, and (iii) adhesion of high-spired-Subulinidae with the trichomes trimmed or left intact on two of the three orchid species.

## Materials and methods

The authors hereby state that all plant and snail material from Bogor Botanic Gardens, Bali Botanic Gardens, Cibodas Botanic Gardens, and the Manusela National Park on Seram was collected under the approval of the Ministry of Research, Technology and Higher Education with permit number IMI.2.GR.01.06.03.1437ES.315) of July 17–31, 2018. No animals were harmed in the making of this article.

### Snails and orchids

**Fig 1:**
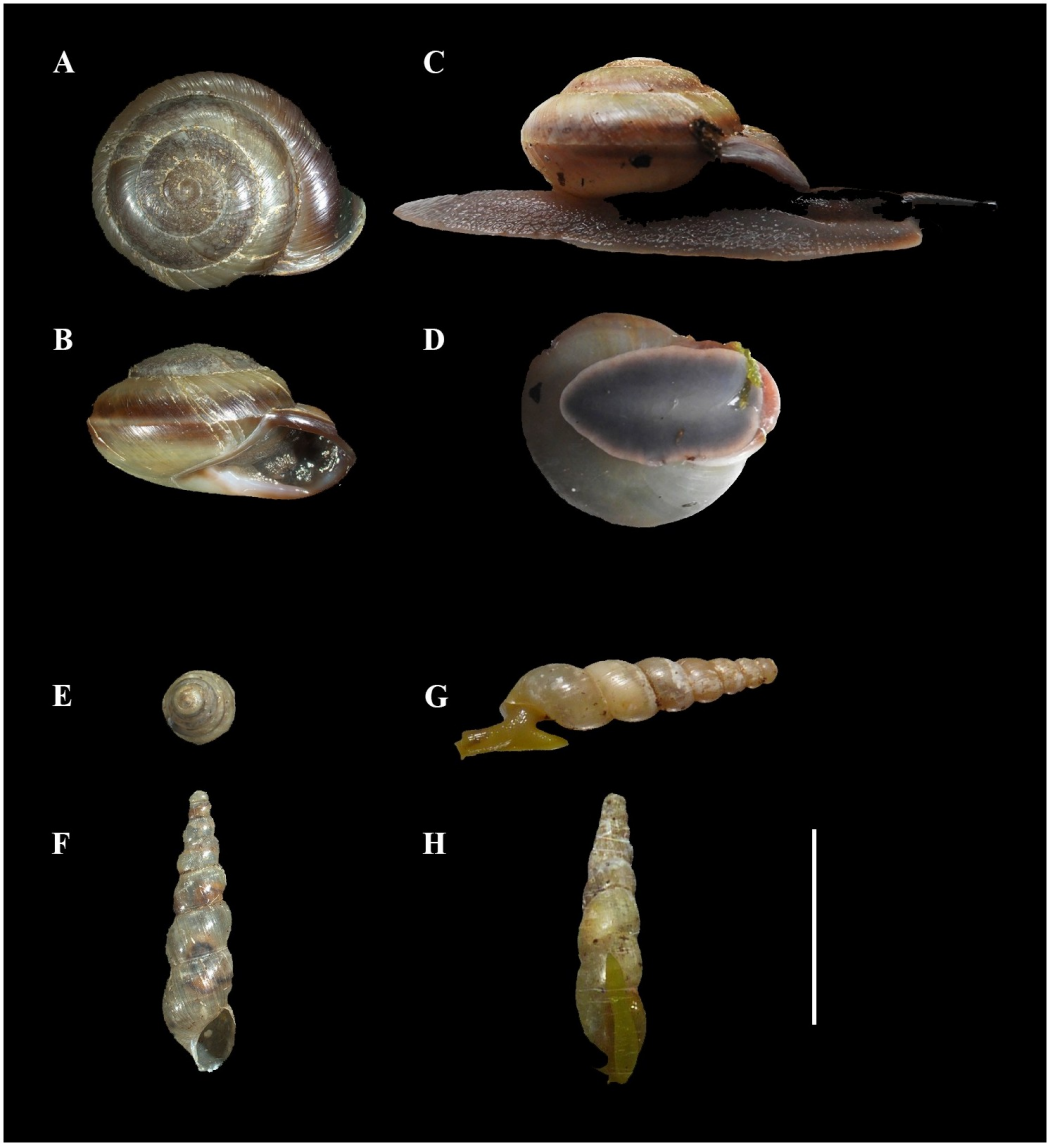
Different views of shells and live snails of the two species used in this study, *Pleurodonte isabella* (A-D) and *Subulina octona* (E-H) to illustrate the species differences in size and shape. A, E, apical view of shells; B, F, apertural view of shells; C, G, extended (crawling) snails in lateral view; D, H, snail soles (crawling surface) attached to glass plate. Scale bar = 10 mm. Photographs by AJdW.

We observed snail species with long and short spired shells consuming orchids in the field and in cultivation (Tables 1 and 2). To represent both groups, and for practical reasons, we chose to work with (sub)adults of *Subulina octona* (Subulinidae) and *Pleurodonte isabella* (Pleurodontidae). *Subulina octona* has a slender high spired shell; the crawling animal’s foot surface, the sole, is elongate and narrow; *P*.*isabella’*s shell is much more globose and the foot of the crawling animal has a much wider sole surface (Fig 1). Live *Subulina octona* snails were collected from the greenhouses of plant breeder *Elstgeest potplanten* by placing traps, consisting of bricks with fresh cucumber slices underneath, below tables on which orchids were stowed. Live *Pleurodonte isabella* snails were purchased from a snail shop in Rotterdam (http://www.slakkenshop.nl). Live animals were placed in plastic tubes with a hole punched in the lid for fresh air and fed with daily refreshed cucumber or lettuce *ad libitum* for the duration of the adhesion experiments. The snails were kept at room temperature in the laboratory. Both species of snails readily consumed leaves from any of the three orchid species investigated.

**Table 1:** Overview of different characteristics and epicuticular properties of three species of orchids investigated in this study. The ranges in *T*. *ferox* represent the ab/adaxial side and the ab/adaxial side respectively.

Orchid species	Epicuticular properties of leaves					
	Trichome type	Trichome location	Length (mm)	Density (per mm2)	Diameter (mm)	Lignin content
***Calanthe triplicata***	glandular	present on abaxial side	0.084±0.01	20±3	0.01	absent
***Dendrochilum pallidiflavens***	glandular	present on abaxial side	0.04±0.006	16±4	0.02	absent
***Trichotosia ferox***	non glandular lignified	present on both sides	0.18± 0.04 1.0± 0.2	8±2–14±2	0.02–0.03	Present

**Table 2:** Details of herbivorous snails and orchids that were found together, both in cultivation and in the field in Indonesia.

Herbivore and orchid species	Localities in cultivation and natural habitat
***Calanthe triplicata***	Manusela National Park, Seram islands, Moluccas, Indonesia
*Curvella* sp. (Subulinidae)	Cibodas Botanic Gardens, Java, Indonesia
*Subulina octona* (Subulinidae)	Kampung Loa Loa, Seram islands, Moluccas, Indonesia
*Ariophanta* sp. (Ariophantidae)	
***Trichotosia ferox***	Manusela National Park, Seram islands, Moluccas, Indonesia
*Leptopoma* sp. (Cyclophoridae)	
***Dendrochilum pallidiflavens***	Cibodas Botanic Gardens, Java, Indonesia
*Curvella* sp. (Subulinidae)	

We measured total mass, total surface in lateral view of the shell, and sole surface when viewed from below for both species of snails for a subset of 15–18 mature individuals per species from different size classes. To enable a more distinct comparison between both species, a size class was determined based on discrete gaps found in the measurements. For each weight class we used three individuals. The weight classes for *Subulina octona* were 1 (0.050–0.56 g), 2 (0.070–0.076 g), 3 (0.080–0.086 g), 4 (0.09–0.096 g) and 5 (0.10–0.16 g). The weight classes for *Pleurodonte isabella* were 1 (2.38–2.39 g), 2 (2.48–2.6 g), 3 (2.71–2.73 g), 4 (2.80–2.84 g), 5 (3.03–3.18 g), and 6 (3.24–3.37 g). Body mass was measured using a ProScale weighing scale. The lateral projected area was measured from photographs, and sole surface area was measured from photographs of snails moving actively on a glass surface using ImageJ [33]. From these data, we used the correlation between body mass and lateral view area and sole pad to estimate the lateral view area and sole pad based on body mass for all specimens.

The herbivores investigated were a representative selection of snails eating from the leaves of three different orchid species: *Calanthe triplicata* (Willemet) Ames (subfamily Epidendroideae, subtribe Collabiinae) and *Trichotosia ferox* Blume (subfamily Epidendroideae, subtribe Eriinae), both recorded and observed in Manusela National Park in Seram, Indonesia in lower montane rainforest, between 1195–1272 m asl); and *Dendrochilum pallidiflavens* Blume subfamily Epidendroideae, subtribe Coelogyninae) growing on Mount Salak in Java (800–1400 m asl).

### Light microscopy

To investigate chemical properties, freshly harvested leaf samples of plants reared in greenhouses were processed into microscopic slides to detect lignin, polysaccharides, carbohydrates, and calcium following protocols of Sheehan and Hrapchak [34] and Dashek [35]. Leaf samples were first embedded in Paraffin Paraplast^®^ Plus (Kendall Health Care Products, Japan) by rinsing the fixed samples in water and dehydrating them in a series of ethanol: xylene solutions. Then, they were stored in xylene for eight hours, infiltrated in Paraffin Paraplast^®^ Plus and placed in an oven at 60°C for one day. Infiltrated samples were solidified and sectioned at 4–8 μm thickness with a Leica RM2265 rotary microtome (USA). Collected paraffin ribbons were laid in a 40-45ºC water bath, mounted on microscope slides, and dried on a hot plate set at 55ºC overnight. Deparaffinization of samples was performed in a series of xylene:ethanol solutions and the following stains were applied to the paraffin sections: an aqueous solution of Toluidine Blue O (TBO) 1% (w/v) in 1% (w/v) sodium borate for 30 seconds to detect mucins, Etzold’s staining (Basic Fuchsin 10 mg, Safranin 40 mg, Astra Blue 150 mg, Acetic acid 2 ml, and distilled water to complete 100 ml) for 3 min to detect lignin, Periodic Acid-Schiff (PAS) staining for 5 min for the detection of insoluble polysaccharides and starch [36] and von Kossa (Sigma-Aldrich) for 30 min to visualize calcium crystals. All sections were mounted in Entellan^®^ (Merck) after dehydration and examined under an AXIO Imager.M2 microscope with camera and Axiovision software (Zeiss, Cambridge) directly after staining.

### Scanning electron microscopy (SEM)

Fixed leaves were dehydrated for 20 minutes in a series of ethanol solutions (70%-96%-≥99.9%) and twice in fresh acetone ≥99.8%. Critical point drying using ≥99.8% acetone and liquid CO_2_ as exchange fluids was performed in an Automated Critical Point Dryer Leica EM CPD300 (Leica Microsystems, Wetzlar, Germany). The drying protocol included a cooling step at 15°C, 50% stirrer speed with auto version, slow CO_2_ influx in the pressure chamber, with a delay of 120 seconds after influx of CO_2_ and before starting the exchange process, 18 exchange cycles (CO_2_: 99.8% acetone), with a fast (10 s) heating speed and medium (1 min) gas out speed. Dried samples were mounted on stubs with adhesive carbon conductive tabs and sputter-coated with 20 nm of Pt/Pd in a Quorum Q150TS (Quorum Technologies Ltd, East Sussex, United Kingdom) sputter-coater. The resulting samples were observed with a JEOL JSM-7600F Field Emission Scanning Electron Microscope (JEOL Ltd, Tokyo, Japan), at an accelerating voltage of 10 kV.

### Transmission electron microscopy (TEM)

Fresh leaves were fixed in modified Karnovsky fixative (2.5% glutaraldehyde, 2% formaldehyde, in 0.1M sodium cacodylate buffer, pH 7.2) for 3 hours on a turntable and rinsed three times in 0.1M sodium cacodylate buffer (pH 7.4). Post fixation was performed in the dark for at least 2 hours in 2% osmium tetroxide in 0.1M sodium cacodylate buffer and rinsing three times with 0.1M sodium cacodylate buffer (pH 7.4). Fixed samples were stained in the dark for 1 hour in an 3% uranyl acetate solution. The samples were dehydrated in a concentration series of ethanol (30%,50%,70%, 96%, and twice in ≥99.9% ethanol) for 15 minutes, each step in a turntable. The ≥99.9% ethanol was later replaced by acetonitrile in two steps of 15 minutes each. The samples were infiltrated in Epon (21.1% DDSA, 47.5% Embed 812, 29% NMA and 2% BDMA, all from Electron Microscopy Sciences) by submerging them in a mixture of acetonitrile and Epon (2:1, 1:1, 1:2) for 20 minutes for each step. After overnight evaporation of the remaining acetonitrile, the samples were placed in fresh Epon for 3 hours in a turntable at room temperature. Later, moderate vacuum pressure was applied for 20 minutes. The samples were embedded in fresh Epon in plastic molds and polymerized at 60°C for 48 hours. Resulting Epon blocks were trimmed in a rotary microtome with glass knives. Then, ultrathin sections of 95 nm were cut with a Leica EM UC7 ultratome (Leica Microsystems, Wetzlar, Germany), with a diamond knife and mounted on film-coated copper slot grids and post stained with uranyl acetate and lead citrate. Resulting samples were observed and photographed with a JEM-1400 Plus TEM (JEOL Ltd, Tokyo, Japan).

### Attachment forces

**Fig 2:**
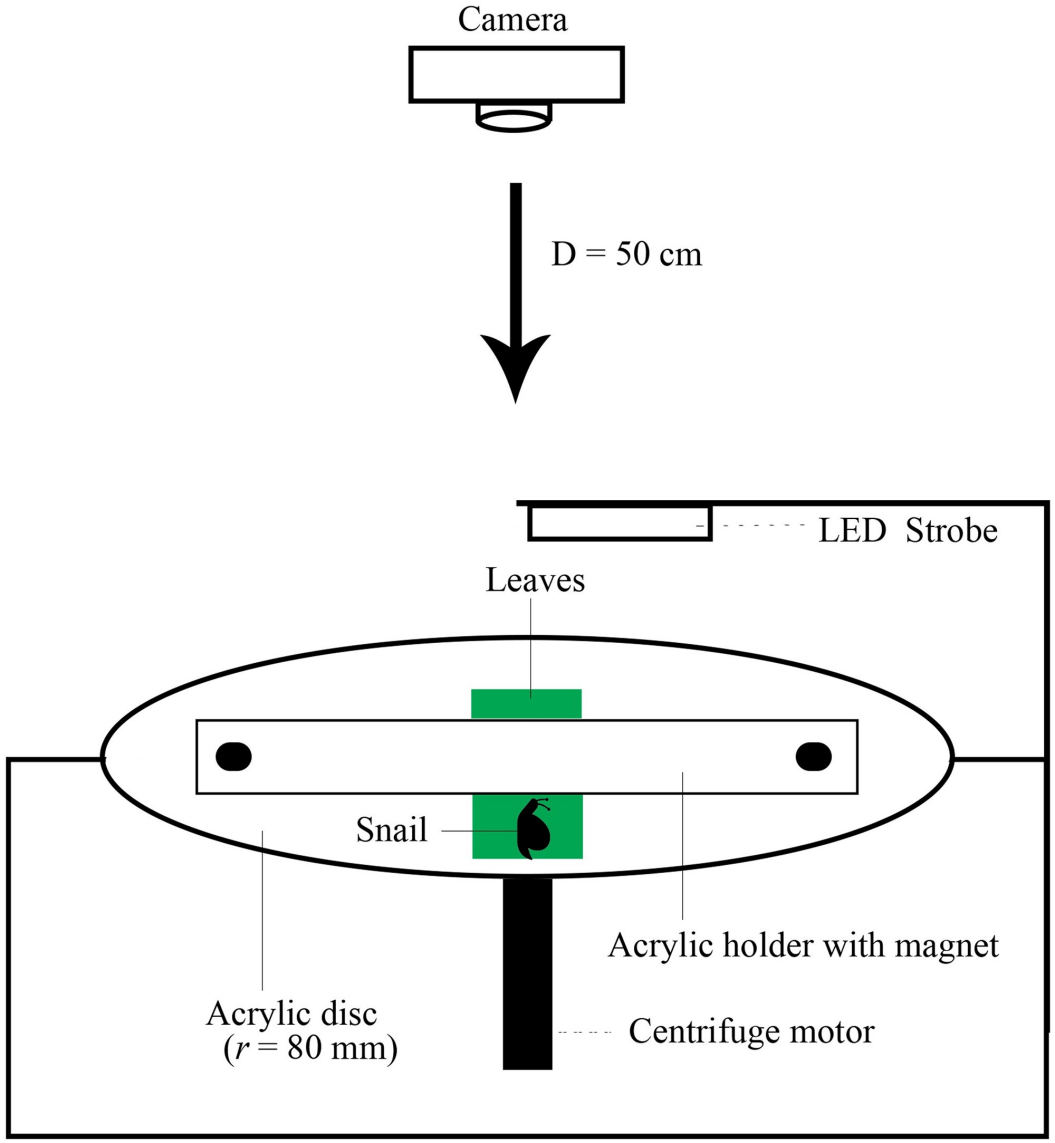
Schematic illustration of the centrifuge method used for measuring surface attachment forces of two different species of herbivorous snails. The snail was placed on a freshly cut piece of orchid leaf that was clipped to an acrylic strip on a horizontally rotating platform, which was accelerated until the snail became detached. A high-speed video camera recorded the run from above and was used to determine the maximum force of detachment. *D*, distance from the camera to the center of the rotating platform; *r*, radius of platform.

To measure the attachment forces of the herbivorous snails, we used a centrifuge technique similar to the method described by Federle et al. [37]. A part of a freshly cut leaf was clipped under two strips of acrylic (80 x 18 mm), held in place by two small magnets, which was placed on a horizontally oriented turntable (radius *r* = 80·mm) mounted on a rotor (Fig 2). We used a strobe light synchronized to the revolutions of the centrifuge through a photoelectric barrier so that a standing image of the snail on the rotating surface could be observed. The centrifuge was filmed from above (distance 50 cm) with a Nikon D5 camera. Cycle duration (in ms) was recorded with an optical tachometer, and the output was displayed on a display on the upper side of the centrifuge housing so that the speed of rotation was visible in the video image.

The snails were placed on a piece of freshly cut orchid leaf, either with the main vein perpendicular to the herbivore or in parallel and on the adaxial (= facing towards stem) or abaxial side (= facing away from stem). Individual herbivorous snails were placed on the turntable facing its centre at a mean distance of 27 mm from the axis of rotation. When the snail had attached itself to the piece of orchid leaf, the centrifuge was slowly accelerated at 0.231cycles*s^-2^. As soon as the snail was dislodged from the leaf, the experiment was stopped. Between every experiment, the orchid leaf fragment was replaced. The force due to centripetal acceleration of the disk (F_c_) was calculated based on the individual body mass in kilograms (*M*_*b*_), the distance of the centre of mass (COM) of the snail from the centre of rotation in meters (*r*) and the cycle duration in seconds (*cycle*) using the formula below:

$$F_{c}=M_{b}r{\left[\frac{2\pi }{cycle}\right]}^{2}$$

The air resistance force due to the drag (F_d_) was calculated from air density (⍴), the lateral projected area (*A*_*l*_) of the snail in m^2^ estimated based on its individual body mass, and the cycle duration in seconds (*cycle*) following the formula below (assuming a drag coefficient of 1):

$$F_{d}=0.5\;\rho A_{l}{\left[(,\frac{2r\pi }{cycle},)\right]}^{2}$$

The magnitude of the total force (F_t_) parallel to the surface of the centrifuge disk needed for removal of the snail was calculated from the centrifugal force and the air drag acting perpendicular to it using the formula below:

$$F_{t}=\sqrt{\left(F_{c}^{2}+F_{d}^{2}\right)}$$

For each snail species, we conducted experiments with ten different animals per orchid species. For each animal, we did three consecutive measurements to not mix naive with experienced animals. We used the average value of each animal for analysis. The animals were allowed to recover for at least 15 minutes between these three measurements. Temperature and humidity were measured during every experiment to correct for their influence on air density [38]. We also calculated the safety factor for each snail species by dividing mean detachment force at each treatment by mean snail body mass.

We applied a trimming treatment to investigate the effect of removing trichomes. The trichomes of *C*. *triplicata* were trimmed by gently scraping the leaves with a sharp blade without damaging the epidermis and subsequently tapping them several times with 3M double tape. We chose a cultivar of *T*. *ferox* that has leaves with a smooth adaxial side and a hairy abaxial side. The subsequent centrifuge experiments were done as described above.

### Statistical analyses

We used REML algorithms to analyze linear mixed models since our data contain both fixed and random factors. To compare attachment performance across individuals that differ in mass and size, we used relative centrifugal force (RCF) rather than absolute force in our statistical analyses, as absolute force depends on the individual’s mass. We did not include the forces induced by air drag or disk acceleration in the statistical analysis, as we found these to be negligible (<1% of the total force). Mean RCF was calculated from the three replicates per specimen, resulting in a single value for each snail. To identify effects of species and leaf side and direction, we then performed a crossed ANOVA using the package LME4 [39] in R version 3.5.0 [40] with log transformed mean RCF as the response variable, snail species and individual as nested independent variables, and orchid species, leaf side, leaf direction and individual as separately nested independent variables. The individual was coded as a random variable. We also tested a more elaborate model that included the log-transformed mass of each specimen (LogMeanRCF ~ (Snail/ (1 | Specimen)) *logMass + Orchid/Side/Direction). The model comparison found the more elaborate model to be no better fit than the simpler model excluding mass (p = 0.737).

To test if surface properties had a significant effect on the attachment RCF, we also tested a model containing the only variables that varied across and within orchid species: trichome length and density. The absence of trichomes was coded as very short trichomes (10^-6^mm), to allow log transformation. The variables snail species, snail specimen, and leaf side and direction were coded as nested random variables. Because we expected the effect of trichome length and density to have an interaction, we initially designed the model with that interaction. As this interaction term had no effect, it was removed. We here present the results of the following mixed model regarding the trichome variables (variations of this model did not significantly alter the outcome): LogMeanRCF ~ (Snail / (1|Specimen)) + Orchid / (1|Side) / (1|Direction) + LogTrichomeLength + LogTrichomeDensity.

## Results

### Epicuticular properties

**Fig 3:**
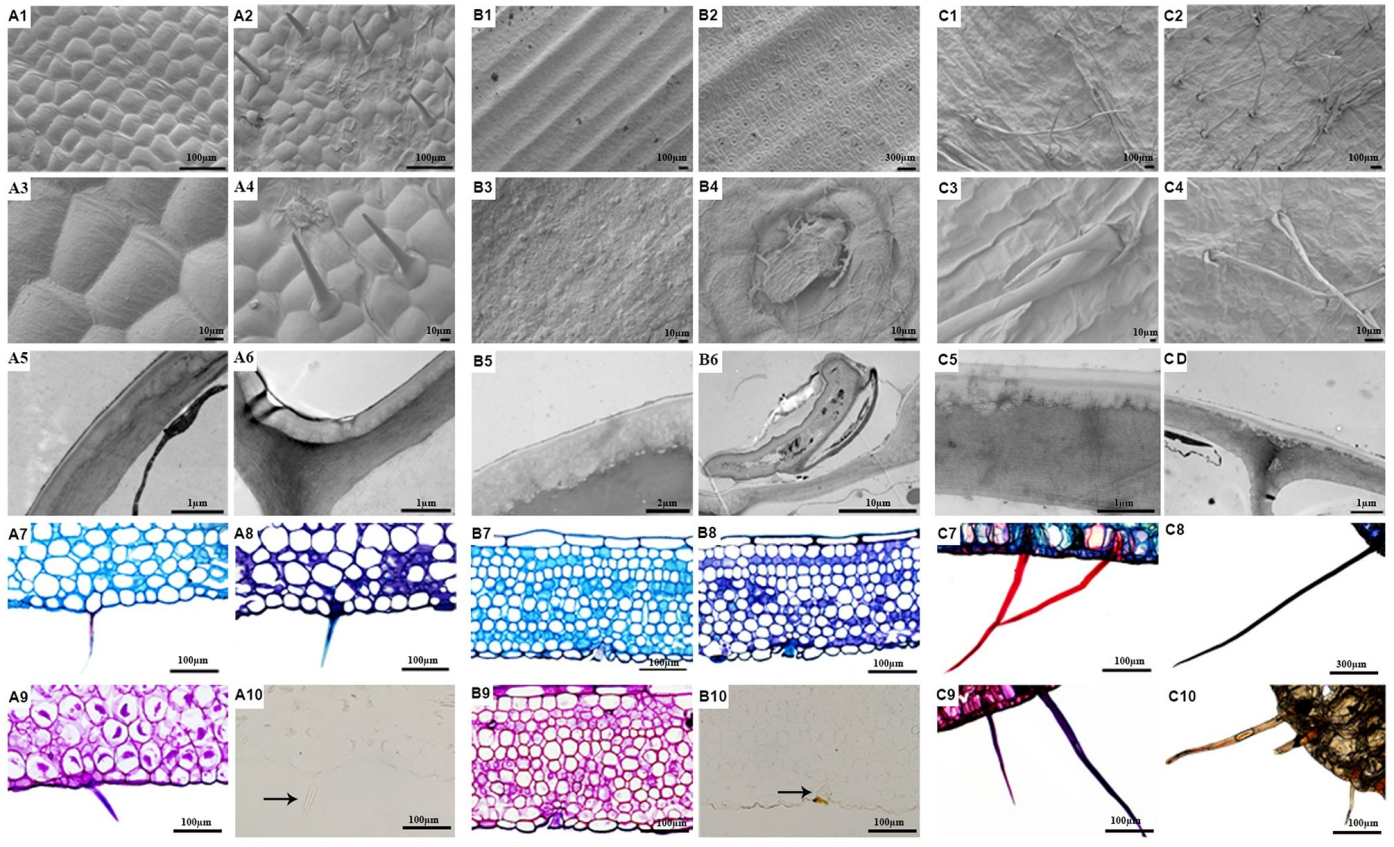
Leaf anatomy and histochemistry of orchid species used in the experiments: A1. Parenchyma cells on adaxial side of leaf of *C*. *triplicata*. A2. Parenchyma cells on abaxial side of leaf of *C*. *triplicata*. A3. Parenchyma cell surface on adaxial side of leaf of *C*. *triplicata*. A4. Trichomes on abaxial side of leaf of *C*. *triplicata*. A5. Section through epidermal surface on adaxial side of leaf of *C*. *triplicata*. A6. Section through epidermal surface on abaxial side of leaf of *C*. *triplicata*. B1. Ornamentation of adaxial side of leaf of *D*. *pallidiflavens*. B2. Ornamentation of abaxial side of leaf of *D*. *pallidiflavens*. B3. Parenchymal cell surface on adaxial side of leaf of *D*. *pallidiflavens*. B4. Trichome on abaxial side of leaf of *D*. *pallidiflavens*. B5. Section through epidermal surface on adaxial side of leaf of *D*. *pallidiflavens*. B6. Trichome basal cell on abaxial side of leaf of *D*. *pallidiflavens*. C1. Surface of parenchymal cells on adaxial side of leaf of *T*. *ferox*. C2. Surface of parenchymal cells on abaxial side of leaf of *T*. *ferox*. C3. Paired trichomes on leaf of *T*. *ferox*. C4. Paired trichomes on leaf of *T*. *ferox*. C5. Section through epidermal surface on adaxial side of leaf of *T*. *ferox*. C6. Section through epidermal surface on abaxial side of leaf of *T*. *ferox*. 7–10: Histochemistry of parenchymal cells of the epidermis of leaves of *C*. *triplicata* (A), *D*. *pallidiflavens* (B), *T*. *ferox* (C). A-C7. Black arrows indicate trichomes. Staining with Etzold (lignin), A-C8. Staining with TBO (proteins), A-C9. Staining with PAS (polysaccharides), A-C10. Staining with van Kossa (calcium).

Detailed SEM, TEM, and LM images of the three different orchid species investigated revealed various epicuticular trichomes of orchid leaf surface (Fig 3). *C*. *triplicata* has short non-glandular trichomes (ca. 0.1 mm long) in a relatively high density (ca. 20/mm^2^) on the abaxial side of the leaves. The leaves of *D*. *pallidiflavens* are covered by short glandular trichomes (ca. 0.5 mm long) on the abaxial side in a relatively low density (ca. 16/mm^2^) that secrete exudates. Leaves of *T*. *ferox* are covered by relatively large (ca. 0.2–1.6 mm long) paired trichomes on both sides. The density of these trichomes is higher on the abaxial (ca. 14/mm^2^) than the adaxial side (ca. 8/mm^2^).

The TEM images revealed the non-glandular trichomes of *C*. *triplicata* and *T*. *ferox* have a thicker cell wall as compared to the glandular trichomes of *D*. *pallidiflavens*. We detected lipid droplets inside the glandular trichomes of *D*. *pallidiflavens*. The different stainings applied revealed that the paired trichomes on the leaves of *T*. *ferox* contain lignin. Proteins and polysaccharides were detected in the trichomes on the leaves of *C*. *triplicata*, *D*. *pallidiflavens*, and *T*. *ferox*. Calcium crystals were detected in the trichomes on the leaves of *T*. *ferox*.

We also measured the gaps between the veins on the leaves of *C*. *triplicata* (7.18–7.59 mm) and *T*. *ferox* (4.48–5.91 mm). The gaps were larger on the leaves of the first species. The larger snail *P*. *isabella* had a wider sole pad (8.38±0.5 mm) as compared with the smaller *S*. *octona* (1.24±0.07 mm). The smaller snail could attach itself in between the veins of both *C*. *triplicata* and *T*. *ferox*. For the larger snail, this was not possible. There were no significant differences in the attachment of both snails in perpendicular and parallel position. This result means that venation did not affect the attachment of the sole pad to the epidermal surface of the leaves.

### Attachment performance

**Fig 4:**
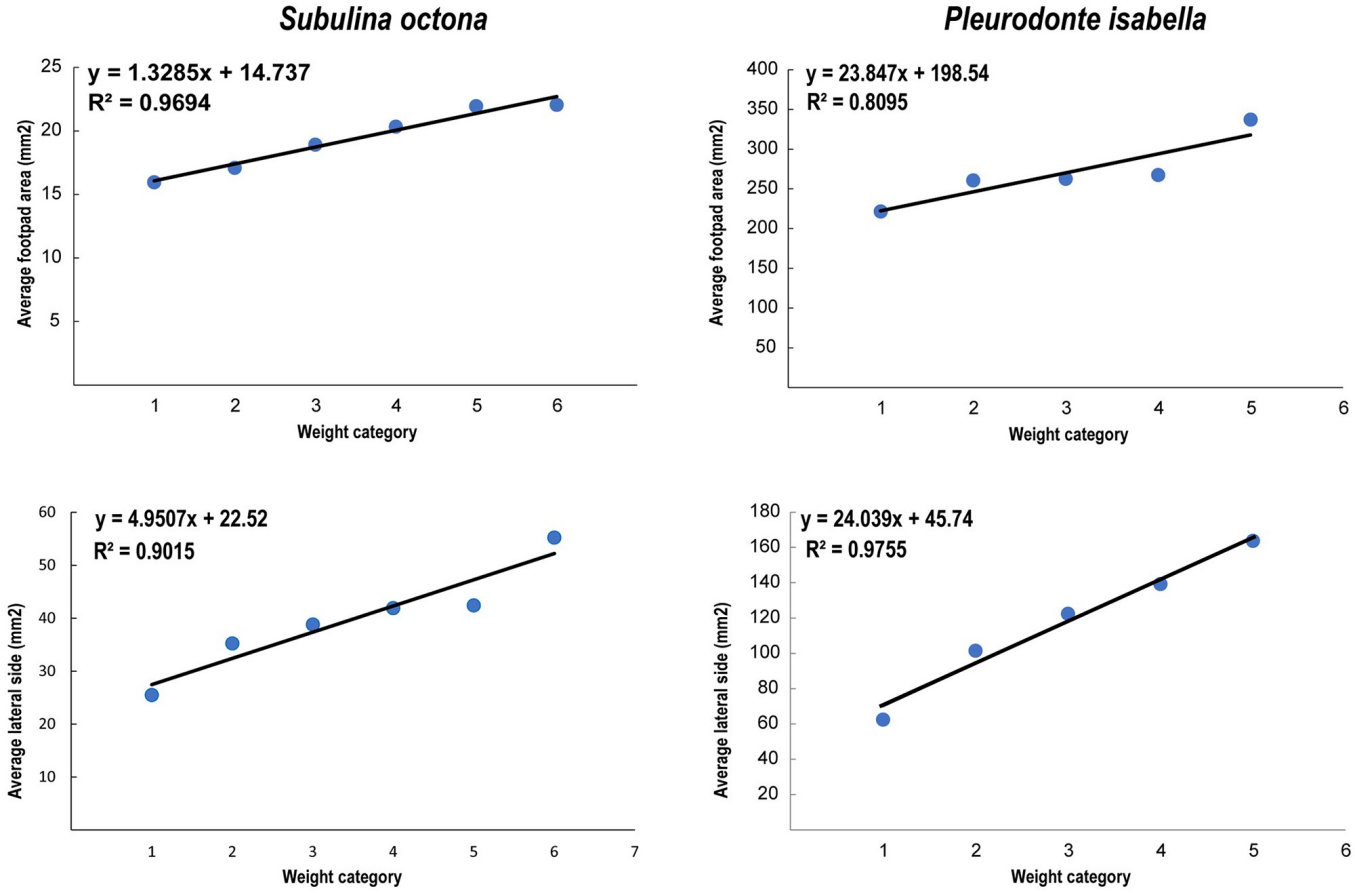
Relationship between A body mass (g) and footpad area (mm^2^) of *S*. *octona* and *P*. *isabella*. B body mass (g) and lateral side area (mm^2^) of *S*. *octona* and *P*. *Isabella*.

The snail species that were used in the centrifuge experiments differed in size, shape, and sole surface area. We compared the weight, the sole surface area and lateral projected area for 3 individuals of each species from different size classes (Fig 4). The mass scaled with sole surface area with an exponent of 1.35 (95% confidence interval: 1.30–1.41) across the two species, which is slightly less than the theoretical isometric value of 1.5. Despite this non-isometric scaling, *P*. *isabella* had 2.5 times more mass (0.0106 g/mm^2^) per unit of sole surface area than *S*. *octona* (0.00424 g/mm^2^). Snail species, orchid species, and the side of the orchid’s leaves, either adaxial or abaxial was found to influence RCF needed for snail removal (see Table 3).

**Table 3:** Analysis of deviance table (Type III Wald F tests with Kenward-Roger df) with mean.

Independent variables	F	Df	Df.res	Pr(>F)
(Intercept)	1017	1	125.96	**<2.2e-16**
Snail Species	32.69	1	119.00	**8.171e-08**
Orchid Species	70.24	1	108.00	**<2.2e-16**
Orchid:Side	3.16	3	108.00	**0.02762**
Orchid:Side:Direction	0.67	6	108.00	0.67053

Dependent variable is the log of the mean of three observations of detachment RCF per snail specimen. Formula: LogMeanRCF ~ (Snail/(1|Specimen)) + Orchid/Side/Direction. P-values that are significant at α = 0.05 are shown in bold face.

We ran the mixed models of mean RCF against predictor variables. Since the difference in fit between the model that included mass and the model that excluded mass was not significant, we here present the results of the simpler mixed model, excluding mass. Where the differences between the models are of interest to understand the role of mass and snail species in attachment, we refer to the more elaborate model, including mass.

The lack of significant difference between the models with and without mass as an independent variable seem to indicate that much of the difference in attachment RCF between the two snail species may be mediated by their difference in mass. However, when including mass in the model, the variance seemed to be divided between species and mass, leading to a marginal non-significant effect for both these variables. Even when including mass, some variance in attachment RCF may still be explained by snail species. The non-isometric scaling of the snails and the potential scaling effects suggested above may contribute to this effect.

Both snail and orchid species have a significant correlation to attachment RCF. Leaf side was also significant in the overall analysis. Particularly the orchids *T*. *ferox* and *D*. *pallidiflavens* showed significant differences in attachment RCF between leaf sides (see Table 4). Of all the orchid species, *T*. *ferox* stood out as different from the other species, as RCF necessary for snail detachment was much lower on this species.

**Table 4:** Linear mixed model fit by REML.

Fixed effects	Estimate	Std. Error	df	t value	Pr(>|t|)
(Intercept)	0.835	0.026	227	31.893	**< 2e-16**
Snail	0.0831	0.0145	227	5.718	**3.38e-08**
*Dendrochilum pallidiflavens*	0.0738	0.0356	227	2.075	0.0391
*Trichotosia ferox*	-0.3228	0.0356	227	-9.069	**<2e-16**
*Calanthe triplicata*: side adaxial	0.0277	0.0356	227	0.780	0.4363
*Dendrochilum pallidiflavens*: side adaxial	-0.0819	0.0356	227	-2.300	0.0223
*Trichotosia ferox*: side adaxial	0.0673	0.0356	227	1.892	0.0597
*Calanthe triplicata*: abaxial: perpendicular	-0.03155	0.0356	227	-0.886	0.3764
*Dendrochilum pallidiflavens*: abaxial: perpendicular	0.00508	0.0356	227	0.143	0.8865
*Trichotosia ferox*: abaxial: perpendicular	-0.0344	0.0356	227	-0.969	0.3337
*Calanthe triplicata*: adaxial: perpendicular	0.0322	0.0356	227	0.907	0.3655
*Dendrochilum pallidiflavens*: adaxial: perpendicular	0.0366	0.0356	227	1.029	0.3045
*Trichotosia ferox*: adaxial: perpendicular	0.0230	0.0356	227	0.649	0.5172

t-tests used the Satterthwaite’s method [‘lmerModLmerTest’]. The dependent variable is the log of the mean of three observations of detachment RCF per snail specimen. P-values that are significant at α = 0.05 are shown in bold.

**Fig 5:**
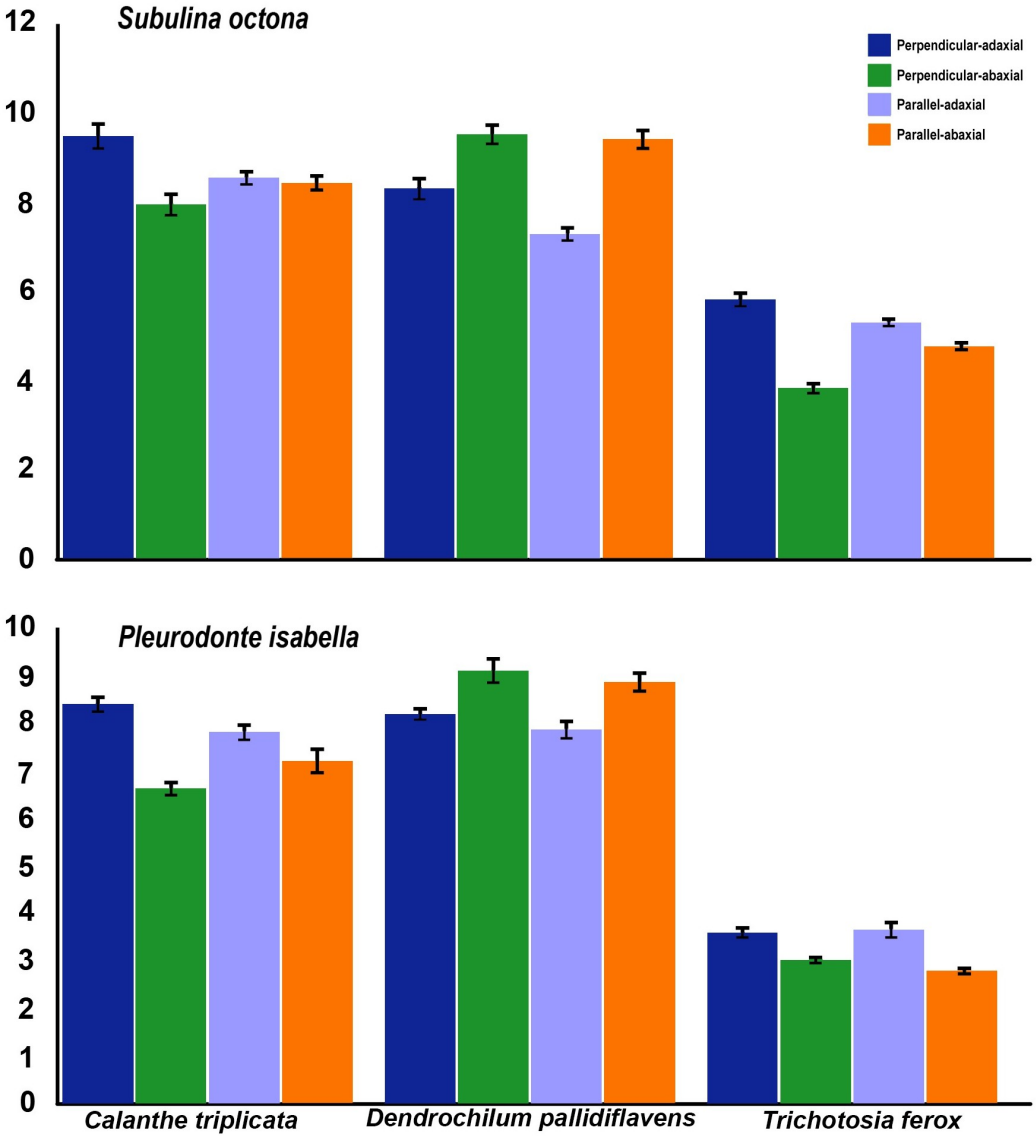
Relative central Force measured (g) of *Subulina octona* and *Pleurodonte isabella* snails on three orchid leaf surfaces. Two positions were tested: perpendicular and parallel to the main veins and adaxial and abaxial side of the leaves. A: *Calanthe triplicata*, B: *Dendrochilum pallidiflavens*, C: *Trichotosia ferox*. Error bars mark the upper and lower 5%.

**Fig 6:**
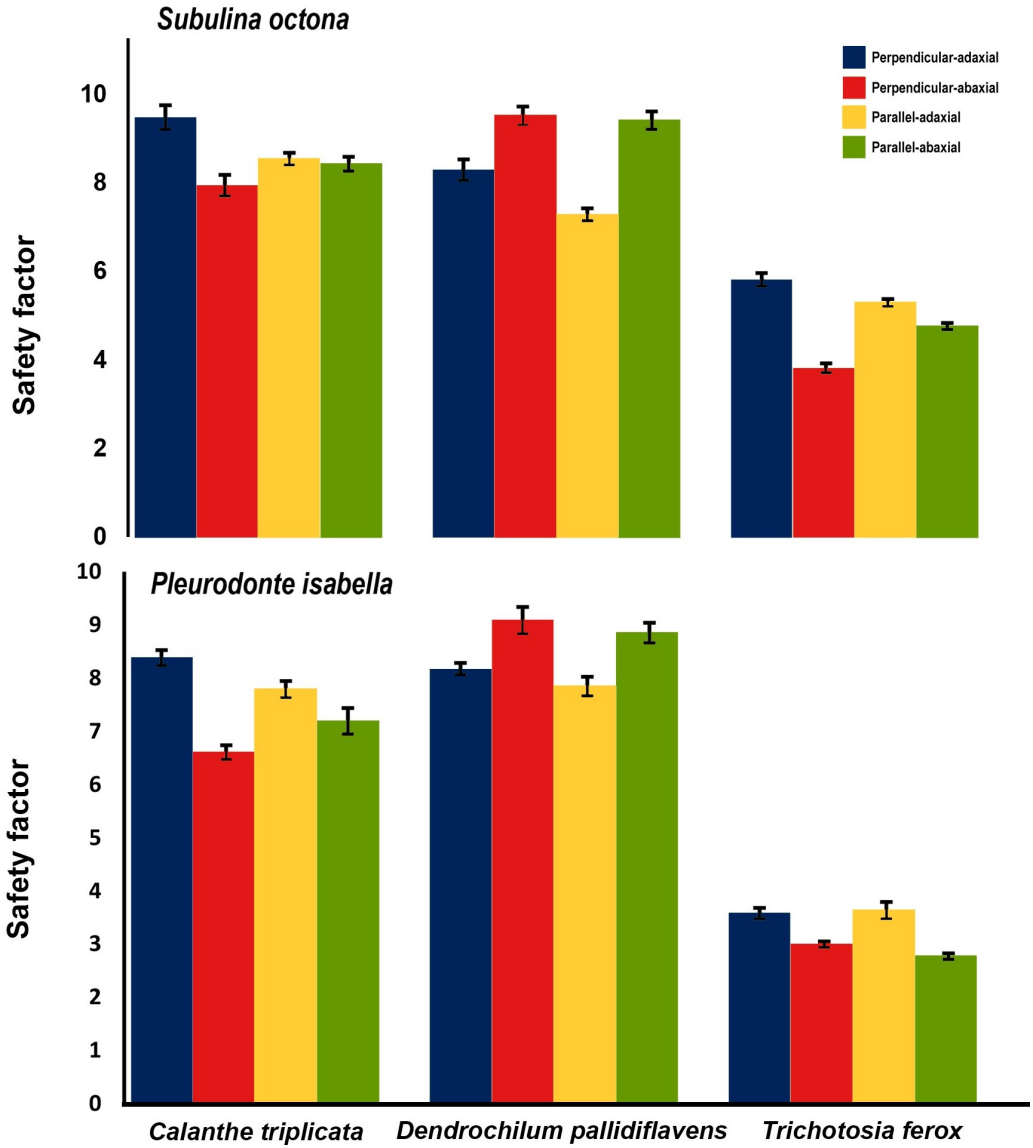
Safety factor of *Subulina octona* and *Pleurodonte isabella* snails on three orchid leaf surfaces. Two positions were tested: perpendicular and parallel to the main veins and adaxial and abaxial side of the leaves. A: *Calanthe triplicata*, B: *Dendrochilum pallidiflavens*, C: *Trichotosia ferox*. Error bars mark the upper and lower 5%.

We found that trichome density and length both had a significant effect on detachment RCF, even taking variables like orchid species and leaf side into account. Higher trichome density had a negative effect on detachment RCF, while trichome length had a positive effect on detachment RCF (Fig 5). The component of force due to centripetal acceleration (CF) was always much larger than that of air resistance (AD; AD_mean_/CF_mean_ = 0.0043). A safety factor calculation showed that both snail species had poor attachment on *T*. *ferox*. (Fig 6 and S1 Table).

**Fig 7:**
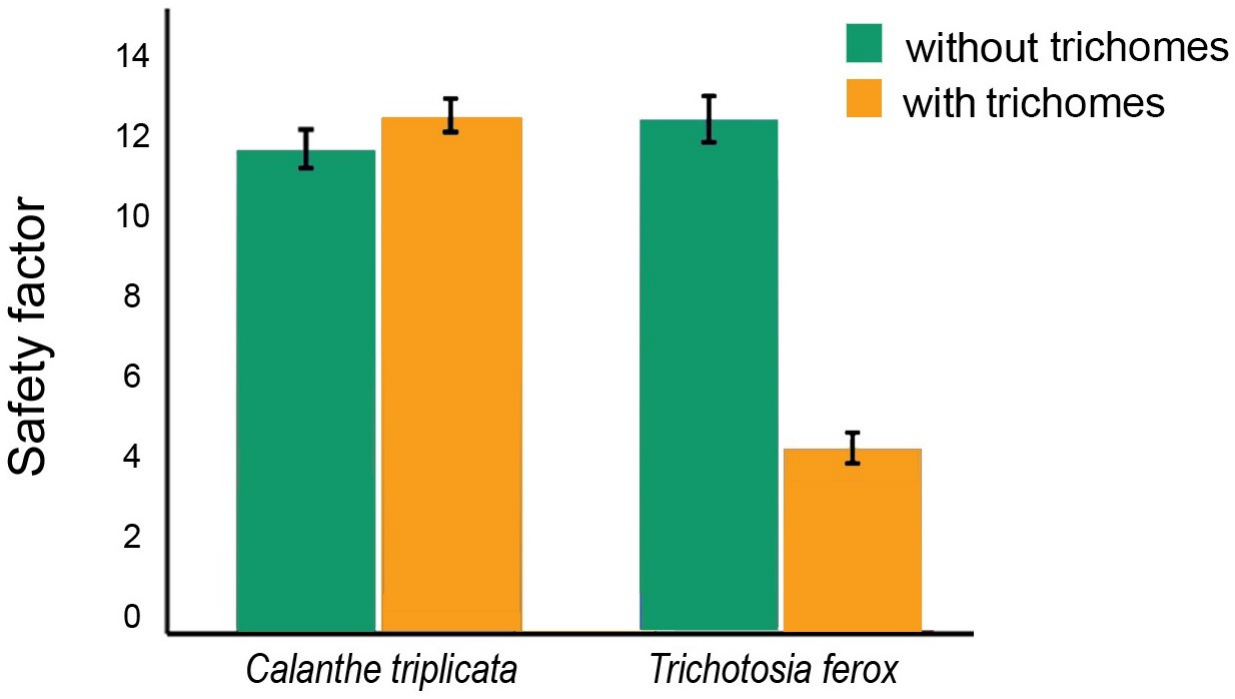
Safety factor of *Subulina octona* snails on trimmed orchid leaf surfaces. Error bars mark the upper and lower 5%.

In addition, we calculated the total force and safety factor for trimmed (smooth) and non-trimmed (hairy) leaves. The result showed there was no significant difference (p = 0.11) in detachment force/RCF of *S*. *octona* on trimmed and untrimmed abaxial leaf sides of *C*. *triplicata*. However, on *T*. *ferox* the attachment of the snails was significantly different (p = 3.31 E-09) between the smooth adaxial and hairy abaxial side with a significantly higher attachment to the smooth adaxial side (Fig 7 and S3 Table).

## Discussion

A higher RCF needed to be applied to remove both snail species from the adaxial side of the leaves of *T*. *ferox*. Leaves of *T*. *ferox* were found to be covered on both sides by trichomes containing lignin, with a slightly higher density of trichomes on the abaxial than the adaxial side. The lower density of trichomes on the adaxial side could explain why the snails were able to hold on longer in the centrifuge experiments. This may be due to the fewer trichomes providing more uncovered leaf surface, and thus a larger area for the footpad of the snails to attach to than on the abaxial leaf side. Stiff trichomes usually contain hard minerals in high concentration, either silica or calcium phosphate [41]. However, no functional studies of fully stiff trichomes have been carried out to date [42]. Some hypotheses of biomineralization of plant trichomes describe the function of these trichomes for plant defense. The tips of stiff trichomes are made up of either silica or calcium phosphate that enables them to damage skins, mucous membranes or exoskeletons of herbivores [43]

The glandular trichomes on the leaves of *D*. *pallidiflavens*, present only in the abaxial side, did not seem to affect the attachment of either species of snails (Table 5). This result suggests that these trichomes do not function as an anti-herbivore defense, a hypothesis further supported by a study of Podroužková et al. [44] who showed that faeces of *Succinea putris* and *Urticicola umbrosus* snails contained glandular trichomes of *Helianthus tuberosus*, indicating that snails readily consume leaves covered by glandular trichomes. The lipid droplets detected in the trichomes of *D*. *pallidiflavens* might, similar to the elaiosomes on the seeds of this orchid species, attract foraging ants. The ants may protect the orchid from herbivores in a mutualistic relationship [45, 46].

**Table 5:** Analysis of deviance table (Type III Wald F tests with Kenward-Roger df) with means.

Independent variables	F	Df	Df.res	Pr(>F)
(Intercept)	161.4	1	11.90	**2.808e-08**
Snail Species	32.97	1	119.0	**7.310e-08**
Orchid Species	100.5	2	113.7	**<2.2E-16**
Trichome Length	13.37	1	113.0	**0.0003896**
Trichome Density	10.13	1	113.1	**0.001881**

Dependent variable is the log of the mean of three observations of detachment RCF per snailspecimen. Formula: LogMeanRCF ~ (Snail / (1|Specimen)) + Orchid / (1|Side) / (1|Direction) + LogTrichomeLength + LogTrichomeDensity. All p-values are significant at α = 0.05 and are shown in bold face.

The adhesive footpad area is the main factor that affects the adhesive force of climbing animals such as herbivorous snails [47, 48]. The production of mucus as an adhesive substance enables snails to traverse various surfaces [28, 49]. *Pleurodonte isabella* did detach at a lower RCF than *Subulina octona*. This may be attributed to the scaling of footpad area to mass, which results in more mass per unit of footpad area as a snail gets larger. Perhaps to partially correct for this problem, the larger species of snails had a slightly larger footpad-to-mass ratio compared to what would be expected under isometric scaling. Isometric scaling describes the condition where objects of different size share the same shape, whereas the relationship between two size measures fits a power function with a particular scaling exponent [50]. Nonetheless, the larger species of snails (*P*. *isabella*) still had about 2.5 times more mass per unit foot area. If the snails would all detach at the same foot stress, we would expect the smaller species *S*. *octona* to detach at about 2.5 times the RCF of *P*. *isabella*. This was not the case, as *S*. *octona* detached at 1.37 times the RCF of *P*. *isabella*. This difference in footpad stress may be due to size-related effects of the surfaces of the orchid leaves, a stronger adhesive mucus, or shell shape (ovate versus elongate) and size-related and different sole surface effects of the snails (e.g., elongate versus more oblong shape). The scale-related factors of leaf irregularity, snail sole edge length, mucus layer thickness and physical properties of the mucus could also interact to explain the observed results. This may be an interesting avenue to explore in future research.

We found significant differences in detachment RCF across orchid species. The lowest values were seen on *T*. *ferox*. Both trichome density and length are significant factors affecting detachment RCF. The safety factor results showed that both snails attached poorly on the lignified trichomes of *T*. *ferox*. Interestingly, increasing trichome density decreased attachment, but increasing trichome length increased attachment. Trichome density was earlier shown to have a negative effect on ovipositional behavior, feeding and larval nutrition of insect pests [51]. Increasing herbivore attachment with increasing trichome length was also recorded by Voigt et al. [52] for *Dicyphus errans* bugs on the surface of *Brassica oleracea* leaves, with a significant positive correlation between force and both trichome length and diameter. According to these authors, the trichomes provide extra grasp for the claws, thus enabling a stronger attachment of the bug. Expanding this study to more orchid species with relatively long trichomes might provide more insights in the overall effect of trichome length on snail detachment.

Trichomes may reduce wet adhesion by creating large asperities on a surface. A wet adhesion mechanism is created by producing a thin fluid layer that creates capillary and viscosity forces between a pad and the surface [53]. The texture of the attachment surface is an important factor for this mechanism. A study by Crawford *et al*. [54] revealed that the wet adhesion of tree frogs was strong on a smooth surface or a surface with small asperities (< 10 μm) but not on a surface with large asperities. This might be explained by insufficient production of fluid to fill the space between the large asperities and creating air bubbles that reduce the attachment to the surface. Alternatively, the large asperities may increase the drainage of fluid from the pad. Trichomes could thus disrupt wet adhesion of snails and other herbivores to leaf surfaces. Other studies on plant trichomes showed a negative correlation between trichome density and damage caused by insect herbivores [51, 55]. Some snail species like *Succinea putris* and *Urticula umbrosus* prefer to eat leaves with dense trichomes, while others, like *Arion* spp. prefer glabrous leaves [44, 56].

Whether leaf surface ornamentation indeed helps plants to free themselves of snail herbivores, depends of course on the forces that may be induced by wind or rain to a leaf. Plant appendages seem to act as damped oscillators [57, 58] with a random input by wind or rain. The two investigated snail species detached at accelerations between 2.0 g and 24.2 g (19.6–237 m/s^2^). Although it seems that at least the lower end of this range can be reached in plants swaying in the wind [59], measurements of g-forces on leaves exposed to natural conditions will be necessary to confirm if this is a viable mechanism for the plants included in this study to eject snails. In terrestrial species, low wind, and a small travel distance needed for the snail to regain its position on the plant may make this mechanism less viable. In epiphytic species, however, loss of footing by the snail may result in it tumbling several meters to the ground, with a lower likelihood of the snail returning to the same plant.

Our study is the first to quantify adhesion forces of herbivores on orchids. Our measurements provide a first estimate of total forces needed to detach herbivorous snails from orchid leaves. A high density of lignin containing trichomes were found to be effective in inducing loss of footing of the snails, possibly due to reduction of the contact area of the sole. Of the leaf epicuticular compounds detected with staining in our study, cutin, lignin, lipids, and polysaccharides such as cellulose are hydrophobic [60, 61] whereas one particular group of carbohydrates, e.g., sugars, are lipophobic. Further studies on the hydrophilic and hydrophobic capacities of the epicuticular protective structures of the leaves investigated here, next to their chemistry and nanostructures as detected for several other plant species might reveal additional details by which orchids protect themselves against snail herbivores. This might ultimately contribute to the design of a bio-coating with sufficient anti-adhesive properties. Alternatively, the attachment ability of snail pests can be decreased by the selection of orchid individuals with a relatively high amount of leaf trichomes to develop new cultivars and improve *ex situ* conservation of endangered species in botanic gardens.

## Supporting information

S1 Table Centrifuge experiments of three orchid species with different type of trichomes on the epicuticular leaf. (XLSX)

S2 Table Mean detachment RCF and force for each combination of factors. (PDF)

S3 Table Centrifuge experiments of two orchid species with trimming treatment of trichomes on the epicuticular leaf. (XLSX)
