# Supplementary material for: Orchids reduce attachment of herbivorous snails with leaf trichomes
Source: PLoS One. 2023 Aug 18;18(8):e0285731. doi: 10.1371/journal.pone.0285731 (PMC10437931; doi:10.1371/journal.pone.0285731)
Supplement: S2 Table — (PDF) [file pone.0285731.s002.pdf]

S2 Table 2. Mean detachment RCF and force for each combination of factors.

**Snail Species**	**Orchid Species**	**Side**	**Direction**	**RCF(G)**	**Force(N)**
S. octona	*C. triplicata*	Adaxial	Perpendicular	9.48	0.00818
S. octona	*C. triplicata*	Adaxial	Parallel	8.54	0.00665
S. octona	*C. triplicata*	Abaxial	Perpendicular	7.95	0.00774
S. octona	*C. triplicata*	Abaxial	Parallel	8.43	0.00703
S. octona	*D. pallidiflavens*	Adaxial	Perpendicular	8.30	0.00699
S. octona	*D. pallidiflavens*	Adaxial	Parallel	7.28	0.00833
S. octona	*D. pallidiflavens*	Abaxial	Perpendicular	9.52	0.00634
S. octona	*D. pallidiflavens*	Abaxial	Parallel	9.41	0.00824
S. octona	*T. ferox*	Adaxial	Perpendicular	5.81	0.00567
S. octona	*T. ferox*	Adaxial	Parallel	5.30	0.00373
S. octona	*T. ferox*	Abaxial	Perpendicular	3.82	0.00520
S. octona	*T. ferox*	Abaxial	Parallel	4.76	0.00427
P. isabella	*C. triplicata*	Adaxial	Perpendicular	8.38	0.283
P. isabella	*C. triplicata*	Adaxial	Parallel	7.79	0.260
P. isabella	*C. triplicata*	Abaxial	Perpendicular	6.61	0.289
P. isabella	*C. triplicata*	Abaxial	Parallel	7.19	0.278
P. isabella	*D. pallidiflavens*	Adaxial	Perpendicular	8.17	0.275
P. isabella	*D. pallidiflavens*	Adaxial	Parallel	7.85	0.301
P. isabella	*D. pallidiflavens*	Abaxial	Perpendicular	9.09	0.238
P. isabella	*D. pallidiflavens*	Abaxial	Parallel	8.85	0.298
P. isabella	*T. ferox*	Adaxial	Perpendicular	3.58	0.158

P. isabella	*T. ferox*	Adaxial	Parallel	3.64	0.110
P. isabella	*T. ferox*	Abaxial	Perpendicular	3.00	0.124
P. isabella	*T. ferox*	Abaxial	Parallel	2.78	0.0926
